# Comparison of three different prehospital wrapping methods for preventing hypothermia - a crossover study in humans

**DOI:** 10.1186/1757-7241-19-41

**Published:** 2011-06-23

**Authors:** Øyvind Thomassen, Hilde Færevik, Øyvind Østerås, Geir Arne Sunde, Erik Zakariassen, Mariann Sandsund, Jon Kenneth Heltne, Guttorm Brattebø

**Affiliations:** 1 Department of Anaesthesia & Intensive Care, Haukeland University Hospital, Bergen, Norway; 2 Department of Health Research, SINTEF Technology and Society, Trondheim, Norway; 3 Department of Research, Norwegian Air Ambulance Foundation, Drøbak, Norway; 4 Department of Public Health and Primary Health Care, University of Bergen, Bergen, Norway; 5 Department of Medical Sciences, University of Bergen, Bergen, Norway

## Abstract

**Background:**

Accidental hypothermia increases mortality and morbidity in trauma patients. Various methods for insulating and wrapping hypothermic patients are used worldwide. The aim of this study was to compare the thermal insulating effects and comfort of bubble wrap, ambulance blankets / quilts, and Hibler's method, a low-cost method combining a plastic outer layer with an insulating layer.

**Methods:**

Eight volunteers were dressed in moistened clothing, exposed to a cold and windy environment then wrapped using one of the three different insulation methods in random order on three different days. They were rested quietly on their back for 60 minutes in a cold climatic chamber. Skin temperature, rectal temperature, oxygen consumption were measured, and metabolic heat production was calculated. A questionnaire was used for a subjective evaluation of comfort, thermal sensation, and shivering.

**Results:**

Skin temperature was significantly higher 15 minutes after wrapping using Hibler's method compared with wrapping with ambulance blankets / quilts or bubble wrap. There were no differences in core temperature between the three insulating methods. The subjects reported more shivering, they felt colder, were more uncomfortable, and had an increased heat production when using bubble wrap compared with the other two methods. Hibler's method was the volunteers preferred method for preventing hypothermia. Bubble wrap was the least effective insulating method, and seemed to require significantly higher heat production to compensate for increased heat loss.

**Conclusions:**

This study demonstrated that a combination of vapour tight layer and an additional dry insulating layer (Hibler's method) is the most efficient wrapping method to prevent heat loss, as shown by increased skin temperatures, lower metabolic rate and better thermal comfort. This should then be the method of choice when wrapping a wet patient at risk of developing hypothermia in prehospital environments.

## Background

Accidental hypothermia, defined as a body core temperature below 36°C [1], increases mortality and morbidity in trauma patients [2-5]. The reported incidence of hypothermia in trauma patients varies from 1.6-47% [4-7]. The early application of adequate insulation to reduce cold exposure, maintain heat balance, and prevent body core cooling is a key feature and an integrated part of prehospital primary care, particularly to stop post-injury hypothermia in rural areas with prolonged evacuation times [8]. Many different methods and products are used worldwide for insulating and wrapping hypothermic patients, but few studies describe the actual effects of these methods. Recommendations or guidelines for what should be used in the prehospital setting are mostly based on tradition and local experience, not on scientific evidence [9-12], the most commonly used methods being ambulance blankets / quilts (ABQ) in the ambulance services, and bubble wrap (BW) in the air ambulance services. Despite the well established use of BW in the Emergency Medical System (EMS), we were unable to identify any published data showing that this is an effective method of preventing hypothermia.

**Figure 1:**
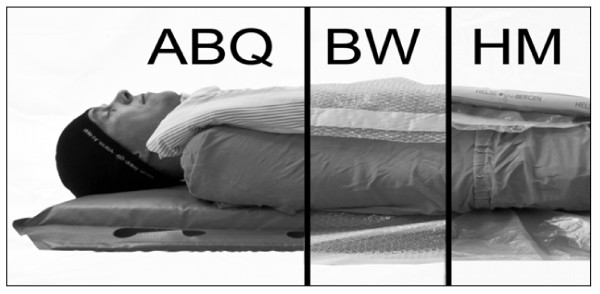
**Wrapping methods**. The three different methods of wrapping the subjects

The thermal properties of different ensembles are determined by their ability to reduce heat exchange through dry and evaporative resistance. Under dry conditions, the insulating capacity is proportional to the thickness of the insulation, while the evaporative resistance becomes more important under wet conditions e.g when patients are wearing wet clothing. The dry insulation values of a range of different insulation materials and methods have been determined by thermal manikins [13], but the effect of wet clothing will significantly increase the evaporative heat loss. To our knowledge, no previous studies have verified the impact of different thermal insulation and evaporative resistance on thermoregulation and body core temperature in humans. Hence, the aim of this study was to compare the thermal insulating effects and comfort of BW and ABQ. We also wanted to compare these results with the so-called Hibler's method (HM), which is a low-cost method combining plastic with an insulation layer (Figure 1). We hypothesised that a combination of a vapour thight layer and a dry insulating layer (HM) is the most efficient in preventing hypothermia when subjects are wearing wet clothing. To evaluate this we measured body temperatures, shivering response and thermal comfort in healthy subjects wearing wet clothing when exposed to a cold anc windy environment.

## Methods

The Regional Research Ethics Committee in Medicine, Central Norway approved the experimental procedure (2009/1181-3). The participants consented to participate and were free to withdraw from the study at any time, without giving any specific explanation.

### Study subjects

Eight healthy, non smoking, male volunteered for the study. They were recruited among students at the Norwegian University of Science and Technology institutions. The subject characteristics were as follows (mean ± SD, n = 7): age, 26.3 ± 6.4 years; height, 181 ± 4 cm; mass, 74.1.± 5.1 kg; body surface area (A_Du_), 54.5 ± 2.2 m^2^; and body fat proportion, 16.0 ± 1.4%. They abstained from physical exercise on the study day, and eating or drinking was not allowed from two hours before the onset of the test until the final measurements were completed. Caffeine and alcohol were not permitted 24 hours prior to the tests. All subjects were submitted to a medical examination before inclusion.

### Testlaboratory

The tests were performed in an EN ISO 17025 accredited laboratory at the Department of Health Research, SINTEF Technology and Society, Trondheim, Norway.

### Experimental protocol

The study was designed to compare the metabolic and thermal responses of healthy humans exposed to three different experimental methods; (1) BW, (2) ABQ, and (3) HM (Figure 1).

The subjects arrived at the preparation room at least one hour before the test. They were fitted with thermistors and heart rate recorders, and rested seated in a chair for 30 minutes at an ambient temperature of 23°C wearing a light kimono in a climate chamber. Moistened test clothing was prepared by leaving the clothing in a plastic bag containing 700 ml water over night in a heating cabinet (25°C). The test subjects then dressed in the preconditioned moist cotton T-shirt, long sleeved shirt, and jeans (total dressing time was 10 min). The subjects then walked into the cold climatic chamber (5°C, and 3 m/s wind) and were placed in a supine position on a 2-mm mattress with their feet towards the fans. After a 30-min initial cooling phase, they were wrapped using one of the three different insulation methods (BW, ABQ, or HM) in random order, on three different days. Wrapping time was set at 10 minutes. Then, the subjects were placed on a standard ambulance mattress (55 mm thick) on the floor. They remained inactive for another 60 min while the measurements were performed. The test was to be terminated immediately if one or more of the skin temperature recordings remained at 10°C or less for more than 20 min, or if their rectal temperature fell below 35°C [14].

### Instrumentation and Measurements

The main outcome measures were mean skin temperature (T_sk_), core temperature (rectal, T_re_), and metabolic heat production (W) (as estimated from O2 uptake measurments (see below), in addition to the participant's subjective evaluation of thermal comfort, thermal sensation, and degree of shivering.

Skin temperature was measured using thermistors (YSI-400 Yellow Springs Instrument, USA, accuracy ± 0.15°C) at 13 predefined locations (forehead, neck, chest, middle back, abdomen, upper- and forearm, hand, front and back of the thigh and calf, and the instep. The average formula of Olesen et al. was used to define mean skin temperatures [15]. Rectal temperature was measured with a thermistor probe (YSI-700, Yellow Springs Instrument, USA, accuracy ± 0.15°C). Data was transferred to a computer for graphical and numerical display of the readings every minute, and processed using TempLog 3.1 (Lab View, National Instruments, Austin TX).

Body fat proportion was calculated using the Durnin and Womersley 4-site skinfold thickness measure [16]. Total body surface in square meters (ADu) was calculated according to DuBois and DuBois [17]. Oxygen consumption (VO_2_) was measured using Oxycon Pro (Jaeger, Hoechberg, Germany, accuracy ± 0.05 L·min^-^¹). VO_2 _(L·min^-^¹) and respiratory exchange ratio (RER) were used to calculate metabolic heat production (W) according to ISO 8996 [18]. RER is assumed to be equal to RQ (respiratory quotient).

A modified, validated questionnaire [19] was used for subjective evaluations of local and overall thermal comfort, thermal sensation, and degree of shivering/sweating. Ratings for thermal comfort were: 1 = comfortable, 2 = slightly uncomfortable, 3 = uncomfortable, and 4 = very uncomfortable. Ratings for thermal sensation were: -5 = extremely cold, - 4 = very cold, -3 = cold, -2 = cool -1 = slightly cold, and (0) = neutral. Ratings for shivering were: 1 = heavily shivering, 2 = moderate shivering, 3 = slight shivering, 4 = no shivering, and 5 = slightly sweating. Subjective evaluations were obtained every 10 min during the experiment.

### Statistical analysis

Power analysis indicated that a minimum of six subjects were needed to detect a between-conditions temperature difference of 0.5°C with 80% statistical power at a α-level of 0.05. The Kolmogorov-Smirnov test was used to test for the normal distribution of continuous variables (T_sk_, T_re_, VO_2_, W). Changes in rectal and mean skin temperatures were assessed by two-way analysis of variance for repeated measures (ANOVA). A within-group study design was used. Skin and core temperature were tested for the effects of time, condition, and interactions between the measures. The temperature data were compared by running a 3-min moving average. Values were analyzed every 5 min. When ANOVA revealed a significant main effect, Student's t-test for pair-wise comparisons was used as a post-hoc test to identify significant differences between the three wrapping conditions. The subjective ratings of thermal comfort, thermal sensation, and degree of shivering were assessed by Student's t-test for paired samples. Results are presented as means with corresponding standard deviations (SD). All differences reported are significant at the 0.05 level. SPSS 16.0 software (SPSS inc. Chicago, USA) and Microsoft Excel (Microsoft Office Excel 2007) were used for the analysis.

## Results

The study protocol was executed as planned. One subject withdrew from the experiments after completing only one test day, and his results are not included in the analysis. One of the rectal probes were dislocated slightly due to movement, and these data are not included in the core temperature statistics. Seven subjects completed all three test series.

### Mean skin temperature (T_sk_)

**Figure 2:**
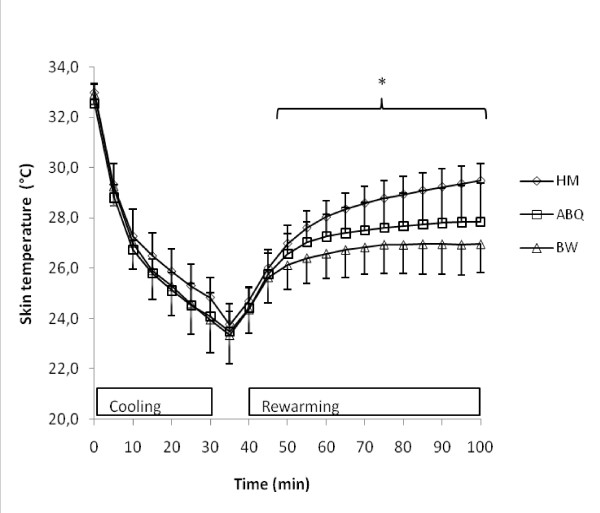
**Change in mean skin temperature (T_sk_)**. Mean skin temperature changes over time. Values are means with SD (n = 7). * Indicates significantly higher T_sk _for the HM method compared with both the ABQ and BW methods (p < 0.05).

T_sk _for the three methods are shown in Figure 2. T_sk _was lower in BW compared to ABQ and HM (p < 0.001) after wrapping. This difference in T_sk _was significant beginning 15 min after wrapping, and remained lower for the duration of the test.

### Core temperature

The analysis showed no significant difference on the T_re _between the three conditions over time. Table 1 shows the core temperature during rest, after cooling, immediately after wrapping, and during rewarming. For all conditions, T_re _did not drop from the resting value during the 30 min cooling period. After wrapping, T_re _decreased significantly for all wrapping methods, and at the end of the rewarming period it was 0.5-0.6°C lower than the initial value after cooling.

**Table 1:** Rectal temperatures during rest, cooling and rewarming

	*Core temperatures (°C) (n = 6)*		
	HM	ABQ	BW
Rest	37.0 ± 0.3	37.0 ± 0.2	37.1 ± 0.2
30 min cooling	37.1 ± 0.3	37.1 ± 0.2	37.2 ± 0.1
Immediate after wrapping	37.0 ± 0.4	37.1 ± 0.2	37.2 ± 0.4
30 min after wrapping	36.8 ± 0.2*	36.9 ± 0.2*	36.9 ± 0.5*
60 min after wrapping	36.5 ± 0.2*	36.6 ± 0.2*	36.6 ± 0.6*

Values are means ± SD (n = 6). *Significant lower T_re _compared to resting value within each condition (P < 0.05)

### Metabolic heat production

**Figure 3:**
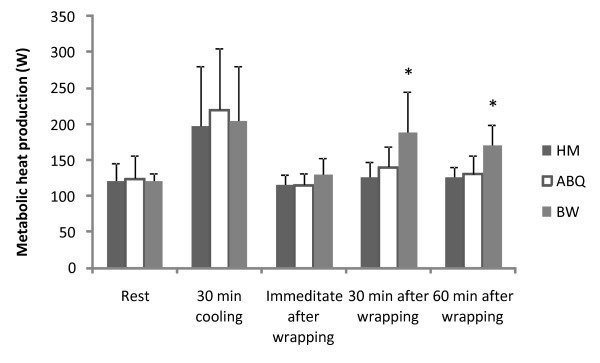
**Metabolic heat production**. Metabolic heat production. Values are means ± SD (n = 7). * Significantly higher heat production by shivering occurred with the BW method compared with either the HM and ABQ methods (P < 0.05)

A significant difference was found between the three methods in metabolic heat production due to shivering over time (Figure 3). The metabolic rate was similar between conditions during rest, and increased 1.6 fold after 30 min of cooling under all conditions. Thirty and sixty minutes after wrapping, the test subjects wrapped in BW had a significantly larger heat production due to shivering, than those wrapped with HM or ABQ, demonstrated in increased metabolic rate.

### Thermal comfort and degree of shivering

**Figure 4:**
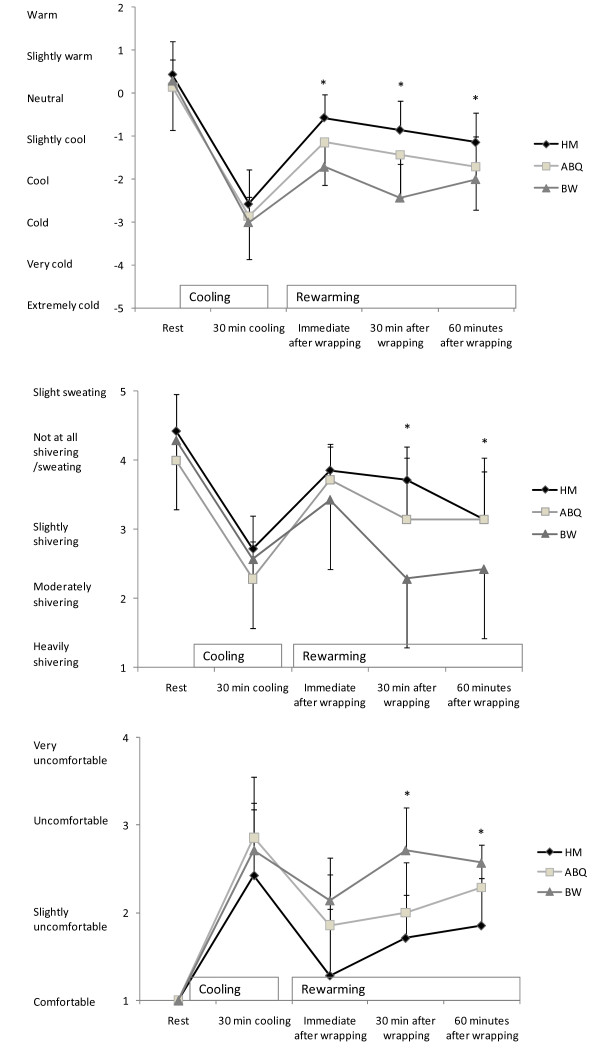
**Shivering, comfort and thermal sensation**. Shivering, comfort and thermal sensation. Values are means ± SD (n = 7). * Significantly colder, more uncomfortable and higher sensation of shivering in condition BW compared to both HM and ABQ (P < 0.05).

Student's T-test for paired samples showed that the subjects felt significantly more uncomfortable, felt colder, and experienced more shivering after being wrapped in BW compared with being wrapped in ABQ and HM (Figure 4).

## Discussion

Hibler's method was the most efficient method to prevent heat loss, shown in higher Tsk and lower shivering response. It was also the preferred wrapping judged by subjective sensation of cold and comfort. BW was the least effective method for preventing hypothermia and seemingly required significantly higher heat production compensate for heat loss. Heat loss is in addition often aggravated due to a combination of exhaustion, clothing, bleeding, entrapment, cold intravenous fluids and/or sedative drugs in the field. The importance of preventing hypothermia and early application of adequate insulation is now one of the cornerstones of prehospital primary care. Interestingly, this priority and management is documented clinically in a recently published article from London HEMS, which led to a change in their practice in the field [7].

### The importance of the material volume

The total heat flux through clothing is commonly considered as the sum of the dry heat transfer and the evaporative heat transfer [20]. Under dry conditions the insulating capacity of different wrapping materials is almost directly proportional to the thickness of the layer (the volume of trapped air in the material) [21]. Therefore, if the patient is dry, and the main heat loss is convection, the choice of material is mainly a matter of local practical characteristics such as usability, price, storage volume, weight and durability. In our study, both HM and ABQ wrapping methods has high thickness and insulation values, but skin temperatures are kept higher in the HM after wrapping. This can only be explained by the evaporative barrier used in the HM. The evaporative barrier hinders the moistness in the wet clothing to be transferred to the outer layers of blankets and quilts, hence reducing the insulative capacity of the material used. In addition, the wet clothing cause increased heat loss by evaporation from the skin resulting in lower skin temperatures in the ABQ condition. This is confirmed by earlier studies demonstrating that evaporative heat loss from the skin and sweating is minimal in cold environments, but could be considerable in the case of wet clothing or wet skin. Under wet conditions, the insulation layers reduces its ability to retain air and thereby reduces thermal insulation. Windy conditions reduce the insulating capacity due to loss of the still outer layer surrounding the material, the compressing effect of the wind and the air permeability of the textiles. Our study confirms this assumption, but also shows that a vapour tight layer of 0,2 mm increases the effect significantly when used in combination with an insulating layer.

### Core temperature, endogenous heat production and comfort

The mean core temperatures were slightly but not significantly lower for HM compared with BW. This can be explained by two factors; firstly, the wrapping inhibited the shivering response, and secondly, the redistribution of the cold peripheral blood from the extremities to the core. The thermoregulatory center stimulates heat production by shivering as a response to the integration of cold information from peripheral and central thermal receptors. The maximal firing rates of cold receptors in the skin are between 17-20°C [22]. When skin temperature increases, the shivering response decreases or ceases entirely. This is supported by the results from the metabolic heat production measures. For all techniques, the shivering rate was reduced when the subjects was wrapped. However the BW method was unable to warm the skin surface sufficiently to inhibit shivering; hence, more intense shivering was experienced with this condition compared to HM and ABQ.

It is likely that the drop in core temperature for the BW group would have been more rapid than for the other two groups, if shivering was inhibited by pharmacological agents or ceased due to trauma, fatigue or severe hypothermia. Light hypothermic patients may be depending on shivering - allowing for spontaneous rewarming - for maintenance core temperature, and caution should be observed when giving sedatives or anaesthesia to shivering patients, in order to prevent further drop in core temperature. The sensation of cold and shivering and thermal comfort was reflecting the lower skin temperatures and shivering response measured. The comfort factor is important when handling patients in the field, and the finding that the subject's feels warmer and more comfortable when wrapped in the HM should be of importance when selecting wrapping method.

### Should bubble wrap still be recommended / used?

Our study shows that a vapour-tight layer like plastic, in combination with an additional insulating layer, is superior to both an ambulance blanket/quilt or bubble wrap used alone. In addition, a blanket/quilt was more effective than bubble wrap. This finding may indicate that there is currently an exaggerated focus on and belief in vapour tight materials used as the sole wrapping method. Nonetheless, we still recommend using bubble wrap as a vapour-tight layer, provided an additional isolating layer is added. The simple, low-cost, and non-invasive nature of Hibler's method makes it a suitable alternative for patients at risk of hypothermia in the prehospital environment. Appropriate measures to avoid cold exposure also include moving the patients into a shelter, removing wet clothing if possible, insulating the patient from the ground, and containing endogenous heat production with an adequate wind- and waterproof outfit/cover.

### Strengths and weaknesses of the study

The design of this study enabled an evaluation of three different prehospital wrapping methods on the metabolic responses in humans with wet clothing. Our study was conducted under standardised conditions in an accredited laboratory, mimicking actual prehospital conditions. Human trials are essential (compared with manikin studies) to verify and determine the impact that different insulation methods could have on human thermoregulation, thermal responses, and body core temperature. If the patient is wet or the insulating material is exposed to rain/snow, then ideally the evaporative resistance, water permeability, and insulation reduction caused by moisture should be considered. Our participants were healthy humans with intact thermoregulatory mechanisms, in contrast to most patients with cold exposure. This may have influenced our results, but to the benefit of reduced heat loss.

The participants were not blinded, and this may have influenced the subjective scorings. However, we do not think this caused any systematic bias since the participants were not informed of the temperature measurements or recordings before or during the tests. Neither did they have any knowledge on the assumed effects of the different treatment methods.

## Conclusions

Prevention and early correction of cold exposure is important because hypothermia is an independent predictor of increased morbidity and mortality in injured patients.

The results of this study show that a combination of a vapour-tight layer and an additional dry insulating layer should be the method of choice when wrapping a hypothermic patient in a prehospital environment.

## Abbreviations

ABQ: ambulance blankets and quilts; BW: Bubble wrap; HM: Hiblers method; Tsk: Mean skin temperature; Tre: Rectal temperature; EMS: Emergency Medical Service.

## Competing interests

The authors declare that they have no competing interests.

## Authors' contributions

OT, JKH and GB conceived and designed the study. GAS and OO contributed in the design and the manuscript writing. HF designed and headed the laboratory testing and, together with MS, performed the calculations. EZ contributed to the manuscript writing. All authors contributed to and approved the writing of the final version of the paper. OT is the guarantor.
